# Supplementary material for: Repurposing tRNAs for nonsense suppression
Source: Nat Commun. 2021 Jun 22;12:3850. doi: 10.1038/s41467-021-24076-x (PMC8219837; doi:10.1038/s41467-021-24076-x)
Supplement: Supplementary file 1 — Supplementary information file [file 41467_2021_24076_MOESM1_ESM.pdf]

SUPPLEMENTARY DATA

Repurposing tRNAs for nonsense suppression

Suki Albers[1], Bertrand Beckert[1], Marco Matthies[2], Chandra Sekhar Mandava[3], Raphael Schuster[4], Carolin Seuring[5], Maria Riedner[4], Suparna Sanyal[3], Andrew E. Torda[2], Daniel N. Wilson[1], Zoya Ignatova[1]*

[1]Institute of Biochemistry and Molecular Biology, University of Hamburg, Hamburg. Germany.

[2]Center for Bioinformatics, University of Hamburg, Hamburg, Germany.

[3]Department of Cell and Molecular Biology, Uppsala University, Uppsala, Sweden.

[4]Institute of Organic Chemistry, University of Hamburg, Hamburg, Germany.

[5]Center for Structural and Systems Biology, Hamburg, Germany.

*Correspondence to: Zoya Ignatova, email: zoya.ignatova@uni-hamburg.de

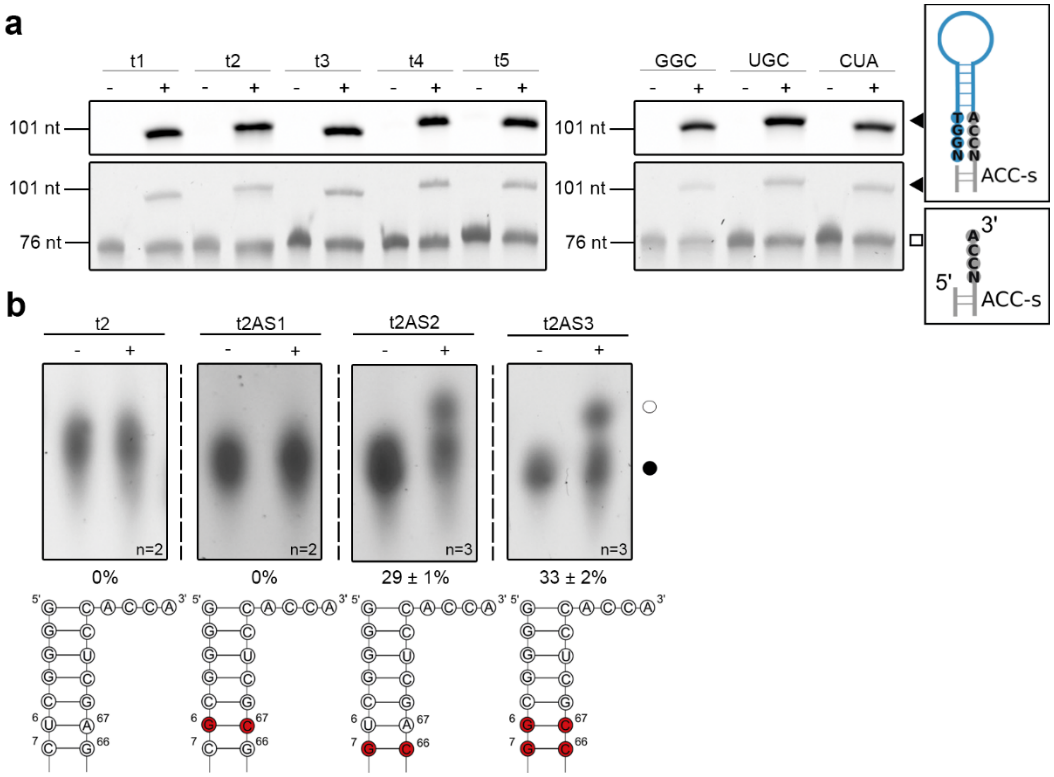

Supplementary Fig. 1. Probing the 3'NCCA intact ends and aminoacylation of the tRNA designs. (**a**) t1-t5 tRNA variants contain similar fractions of intact single-stranded 3'-CCA termini – the prerequisite for aminoacylation. The single-stranded 3'-CCA ends at the end of the acceptor stem (ACC-s) were probed with a Cy3-labeled hairpin oligonucleotide (blue in the schematic) with a complementary 5'-TGGN-3' overhang (+) and compared to *in vitro* transcribed tRNAs (-). tRNAs with ligated oligonucleotide (◄, 101 nt in size) migrate slower than the unligated tRNAs (□, 76 nt in size). tRNAs were detected by fluorescence (upper panel) or SYBR^{TM} Gold Nucleic Acid Stain (lower panel). GGC and UGC denote the anticodon of two natural tRNA^{Ala} isoacceptors and CUA is tRNA^{Ala}(GGC) with exchanged anticodon to decode UAG stop codon. Note that in all samples the oligonucleotide ligates only to a fraction of the tRNAs, i.e. such with intact 3'-CCA ends (lower gel); tRNAs with aberrant ends (i.e. N+1 ends as commonly observed in *in vitro* transcriptions) will appear in the unligated band (lower panel). The experiment serves as a quality check and hence, was performed as a single experiment. (**b**) Aminoacylation of t2 is restored by a single base pair exchange within the acceptor stem. Aminoacylation with Ala catalyzed by *E. coli* AlaRS (+) compared to non-aminoacylated *in vitro* transcribed tRNAs (-). Acylated tRNAs (○) migrate slower compared to non-acylated tRNAs (●). Aminoacylation level are means ± s.d. (n, biologically independent experiments for each designated tRNA). Nucleotide substitutions in the acceptor stem (AS) of t2 are highlighted in red. A single base pair substitution in t2 (C_7-G_{66} to G_7-C_{66}) in t2AS2 raises the aminoacylation level to that of the native tRNA^{Ala} (Fig. 2). Note that both successive changes in t2AS3 render the acceptor stem equal to the acceptor stem of t1 (Supplementary Table 1). Source data are provided as a Source Data file.

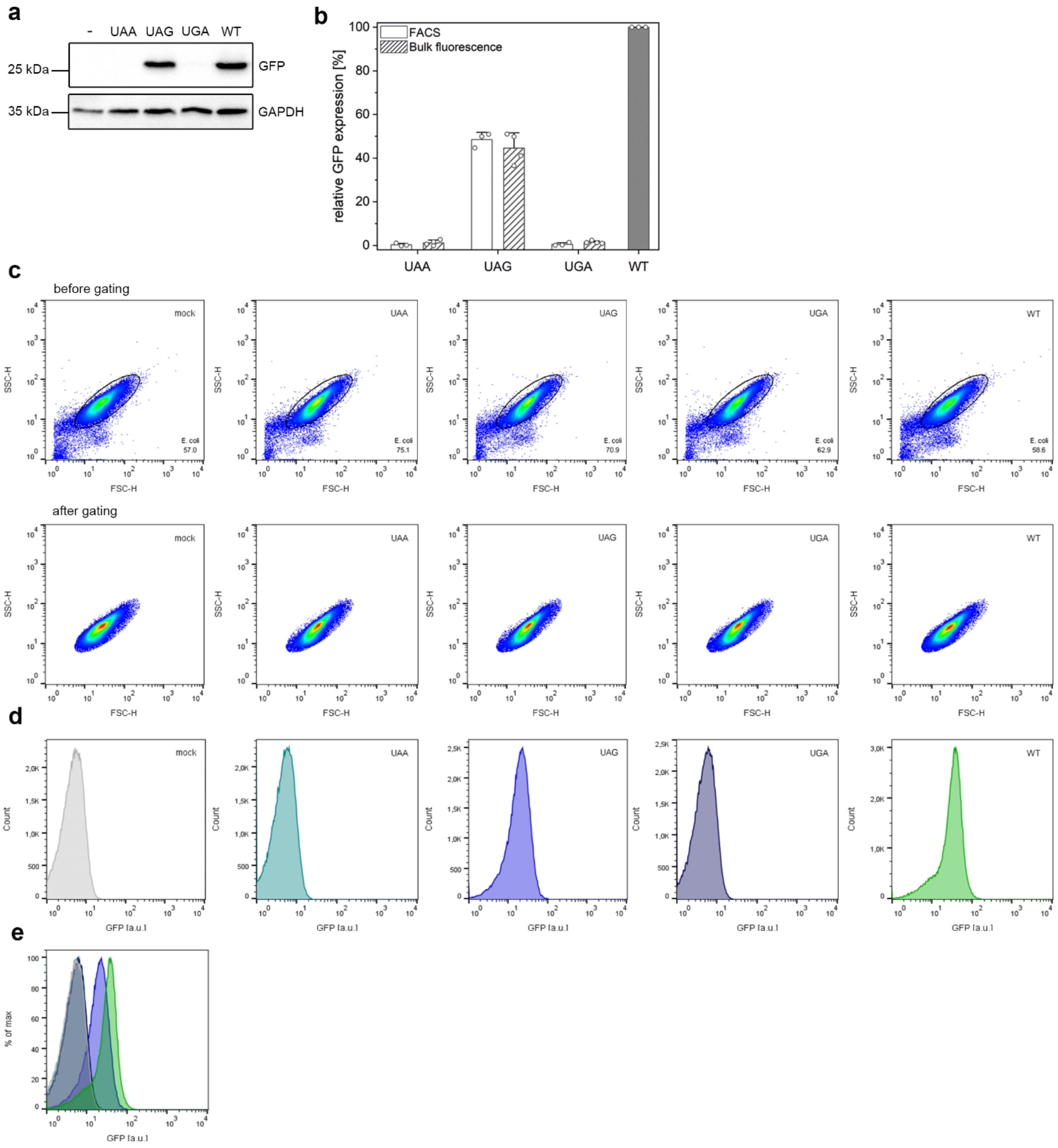

Supplementary Fig. 2. *E. coli* XL1-blue cells express an endogenous suppressor tRNA *supE44* decoding UAG codons. (**a**,**b**) Endogenous readthrough in XL1-blue cells tested with UAA-, UAG- and UGA-containing GFP variant and detected by immunoblotting (**a**) or bulk fluorescence and flow cytometry (**b**). A representative immunoblot is presented in panel a (n=9 biologically independent experiments). -, mock transformation. Bulk fluorescence and flow cytometry signals were normalized to the expression of wildtype GFP (wt, dark gray) whose expression was set to 100%. Data are means ± s.d. for flow cytometry (n=3 biologically

independent experiments) and bulk fluorescence (n=4 biologically independent experiments). (**c**) Representative flow cytometry plots showing side scatter (SSC-H) vs. forward scatter (FSC-H) used to gate mock transformed bacterial sample. The same gate was applied to all samples within one biological replicate. Flow cytometry data are represented as pseudocolor plots. (**d**) Representative GFP fluorescence histograms from the gated populations in panel c. (**e**) Overlay of the representative histograms from panel d. Source data are provided as a Source Data file.

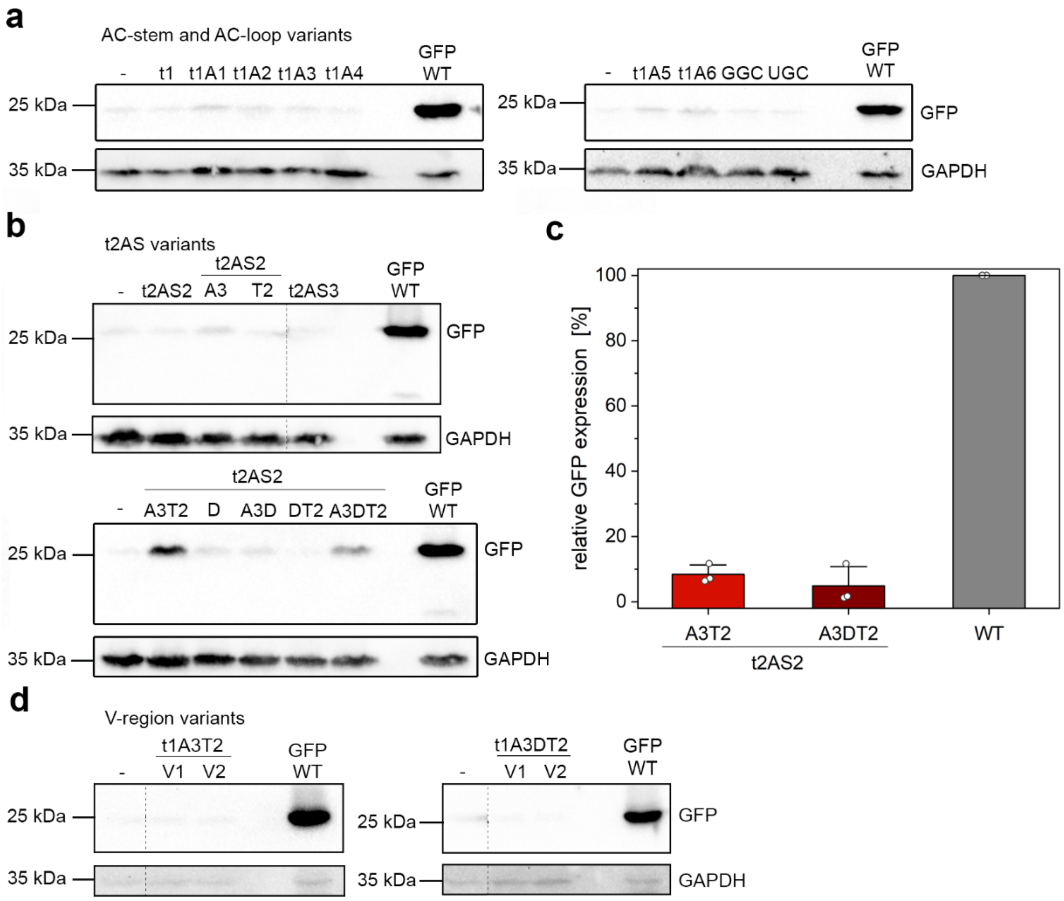

Supplementary Fig. 3. Suppression efficiency of various tRNA designs in *E.coli* XL1-blue determined by readthrough of UGA-containing GFP. (**a**) Representative immunoblot (n=2) of suppression of t1 and its anticodon-edited variants t1A*i*. As control, native $tRNA^{Ala}$ isoacceptors GGC and UGC which do not pair to the UGA codon were used. -, mock transformation. (**b, c**) *In vivo* suppression efficiency of t2AS2, t2AS3 and anticodon-, TΨC- and/or D-region-edited t2AS variants tested in *E. coli* expressing an UGA-containing GFP variant monitored by immunoblotting (**b**), or bulk fluorescence (**c**). Representative immunoblots are present in panel b (n=3 biologically independent experiments); -, mock transformation. The bulk fluorescence in panel c was normalized to the expression of wildtype GFP (wt, dark gray) whose expression was set to 100%. Data are means ± s.d (n=3 biologically independent experiments). (**d**) Representative immunoblot (n=2) of suppression of the V-region-extended variants of t1A3T2 and t1A3DT2. -, mock transformation. Dashed vertical lanes (b, d) denote excised lanes with samples unrelated to this experiment. Source data are provided as a Source Data file.

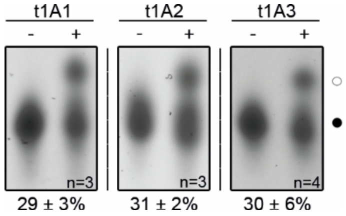

Supplementary Fig. 4. Anticodon-editing does not affect aminoacylation levels. Aminoacylation with Ala catalyzed by *E. coli* AlaRS (+) compared to non-aminoacylated *in vitro* transcribed tRNAs (-). Aminoacyl-tRNAs (○) migrate slower compared to non-acylated tRNAs (●). Aminoacylation level are means ± s.d. (n, biologically independent experiments for each tRNA). Nucleotide substitutions were performed in the anticodon stem and loop of t1 (Fig. 3a). Source data are provided as a Source Data file.

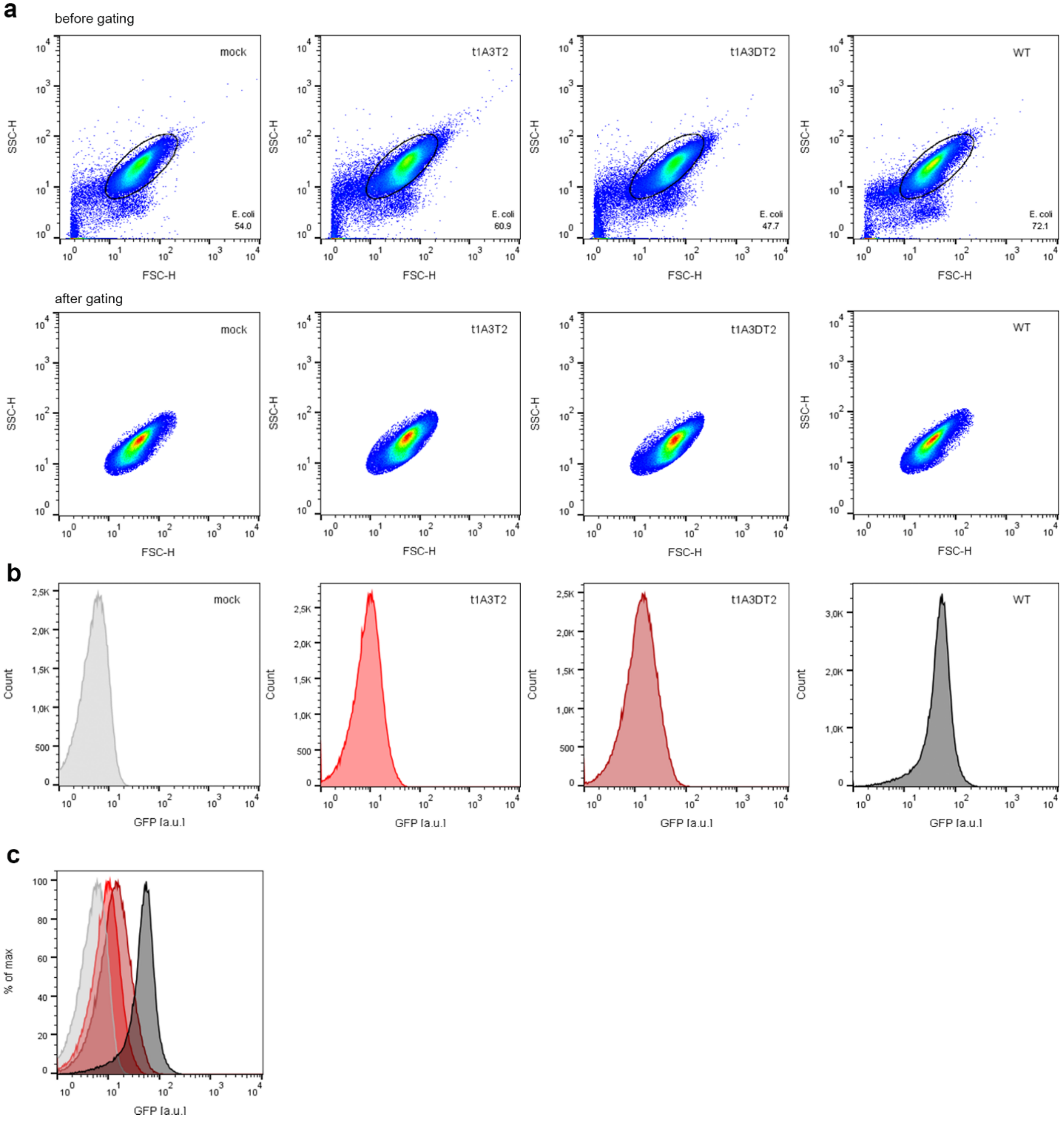

Supplementary Fig. 5. Representative example of gating of the flow cytometry data to Figure 3d. (**a**) Representative flow cytometry plots showing side scatter (SSC-H) vs forward scatter (FSC-H) used to gate intact bacteria in the mock transformed sample. The same gate was applied to all samples within one biological replicate. Flow cytometry data are represented as pseudocolor plots. (**b**) Representative GFP fluorescence histograms from the gated populations in panel a. (**c**) Overlay of the representative histograms from panel b. Source data are provided as a Source Data file.

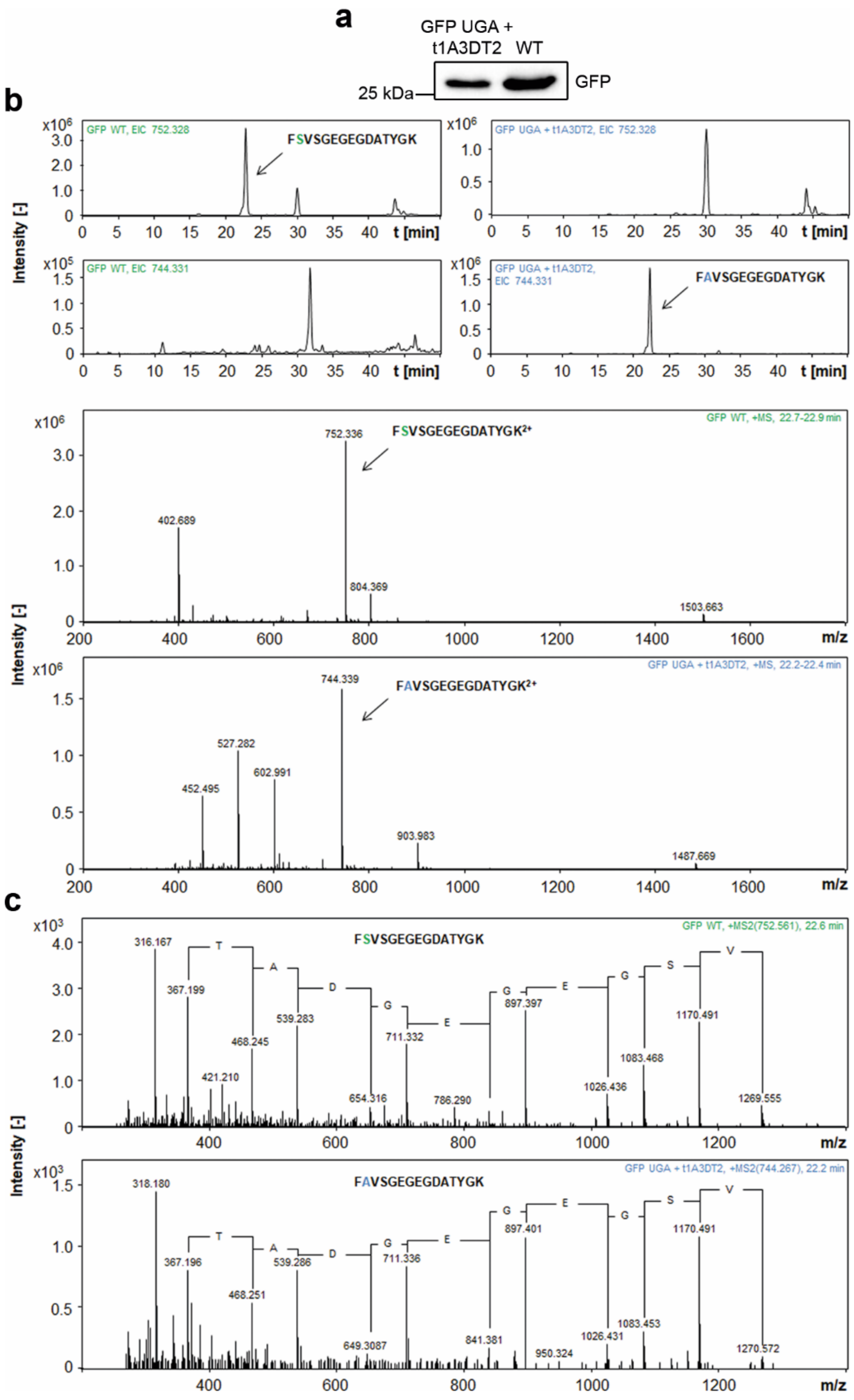

Supplementary Fig. 6. t1A3DT2 incorporates Ala in place of UGA stop codon. (**a**) Affinity purification of wildtype GFP (WT) and the readthrough product of the UGA-containing GFP variant by t1A3DT2 (GFP UGA + t1A3DT2) detected by anti-GFP antibody (Roche). Source data to panel a are provided as a Source Data file. (**b**) LC-ESI-Q-TOF identification of the incorporated amino

acid at the UGA stop codon. Extracted Ion Chromatograms (EICs) of m/z 752.328 and 744.331, corresponding to the GFP-derived peptides. Wildtype GFP amino acid (Ser) is marked in green, the UGA-containing GFP variant readthrough by t1A3DT2 (Ala) in blue. At 22.8 min retention time the intensive m/z-signal of 752.336 corresponds to double-protonated and double-charged GFP-derived peptide F**S**VSGEGEGDATYGK. At 22.3 min retention time an intensive m/z-signal of 744.339 corresponds to double-protonated and double-charged GFP-derived peptide F**A**VSGEGEGDATYGK. (**c**) MS/MS-spectra. The mass difference of the precursors m/z 752.561 and m/z 744.267 corresponds to Ser (wildtype, green) to Ala (stop-codon incorporated amino acid, blue) exchange. The experiment serves as quality check and hence performed as a single experiment.

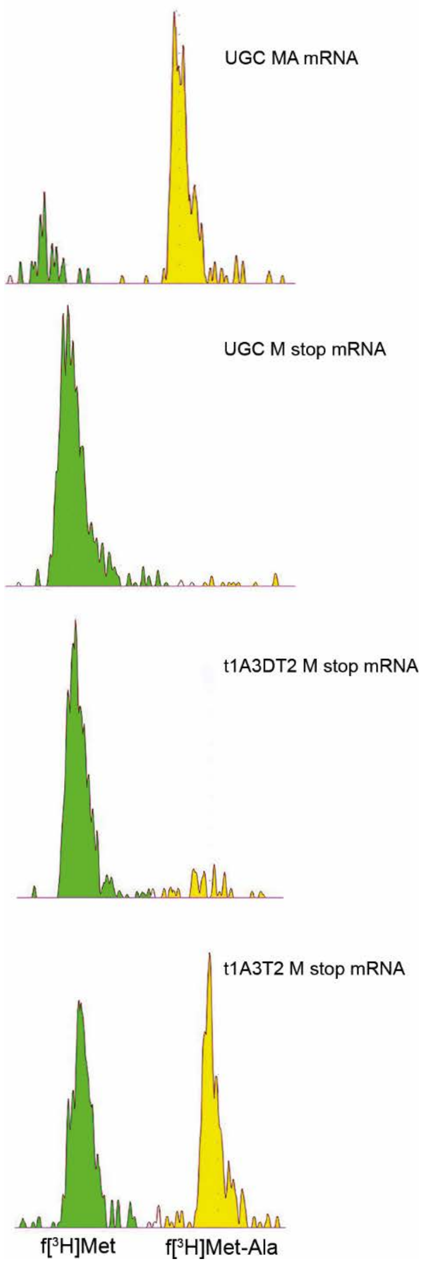

Supplementary Fig. 7. Dipeptide formation with t1A3T2 and t1A3DT2 suppressor tRNAs. RP HPLC chromatogram seperating mono- (f[^{3}H]Met, green) and dipeptides (f[^{3}H]Met-Ala, yellow). Dipeptide formation of t1A3T2 and t1A3DT2 compared to wildtype tRNA^{Ala}(UGC) using either Met-Ala-stop(UAA) (MA) or with Met-stop(UGA) (M stop) mRNAs as templates.

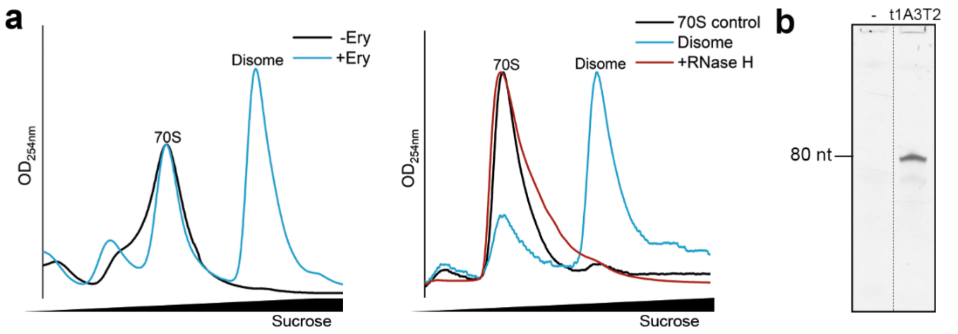

Supplementary Fig. 8. Isolation of t1A3T2 from XL1-blue cells and generation of 70S-UGA complexes. (**a**) Analytical sucrose density (15-60%) gradient profiles of the *in vitro* translation of the 2x ErmCL UGA construct (left panel) performed in the absence (black) or presence of 10 µM erythromycin (Ery; blue) and of the treatment of disomes (right panel) in the absence (blue) or presence of RNase H (red). Isolated 70S monosomes (black) were used as control. RNase H was used to convert disomes into monosomes to avoid orientation bias in the cryo-EM analysis. Source data are provided as a Source Data file. (**b**) Affinity purified t1A3T2 of total RNA isolated from mock transformed cells (-) or XL1-blue cells transformed with pBST NAV2 t1A3T2-tRNA on denaturing PAGE. A biotinylated DNA oligonucleotide complementary to the 3'-end of t1A3T2 was used for the affinity purification. The experiment serves as a quality check and hence, it was performed as a single experiment. Dashed vertical lanes denote the place of excision of lanes with samples unrelated to this experiment. Source data are provided as a Source Data file.

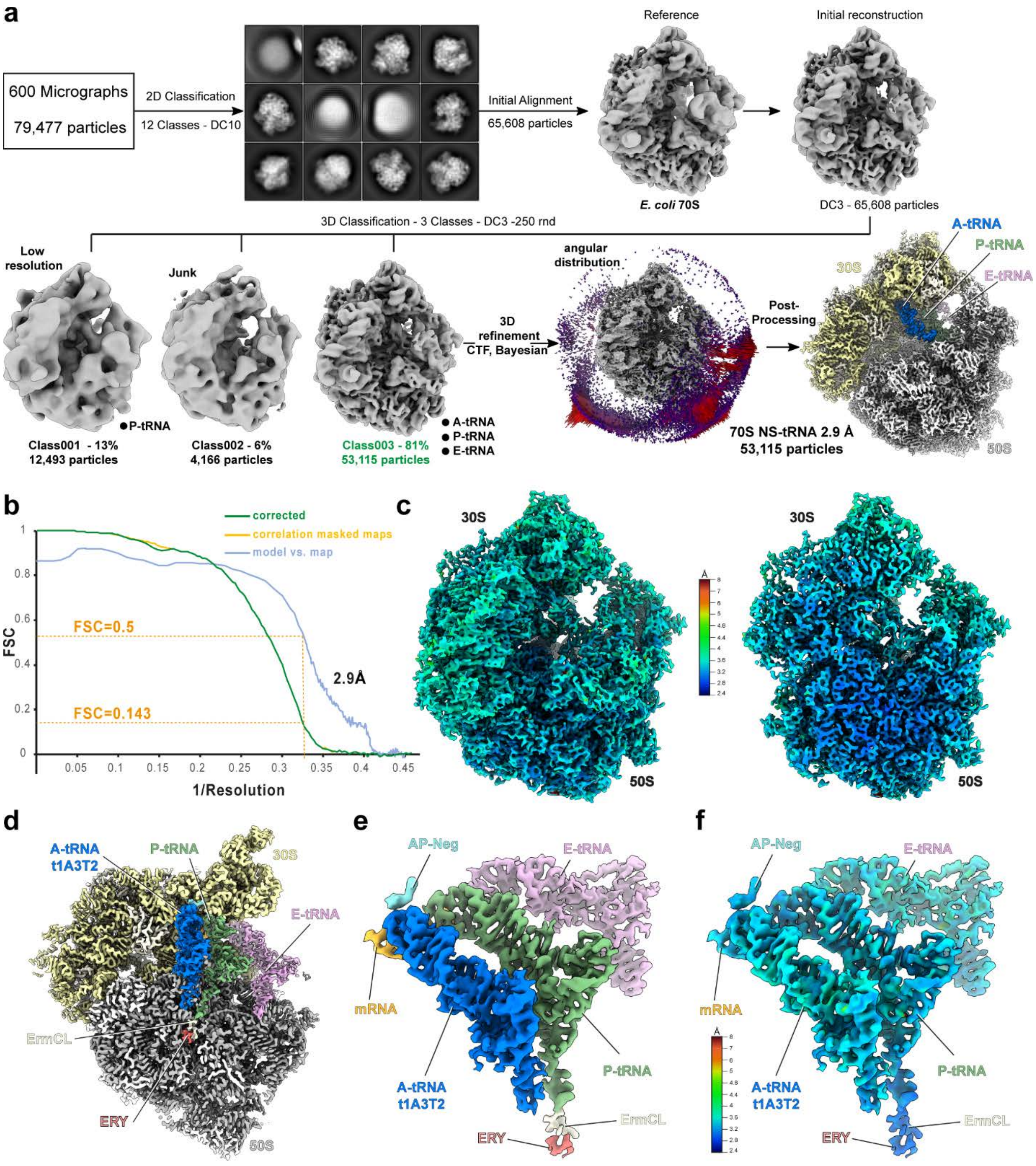

Supplementary Fig. 9. Cryo-EM reconstruction and molecular model of the t1A3T2-tRNA decoding UGA on the ribosome. (**a**) Sorting scheme for the cryo-EM reconstruction of the t1A3T2-UGA-AP-Neg-70S complex. (**b**) Fourier shell correlation (FSC) curves (corrected, green; correlation masked map, yellow and map vs. model, blue) of the final reconstruction of the t1A3T2-UGA-AP-Neg-70S complex indicating an average resolution of 2.9 Å at $FSC_{0.143}$. (**c**) Cryo-EM map of the t1A3T2-UGA-AP-Neg-70S complex colored according to local resolution. (**d**) Overview

of the ErmCL-stalled ribosomal complex with 30S (yellow), 50S (grey), A-tRNA (blue), P-tRNA (green), E-tRNA (pink), ErmCL (beige) and erythromycin (ERY, red). (**e**) Segmented densities for the A-tRNA (blue), P-tRNA (green), E-tRNA (pink), AP-Neg (cyan), mRNA (orange), erythromycin (ERY, red) and the ErmCL-nascent chain (beige). (**f**), As (d), but colored according to local resolution.

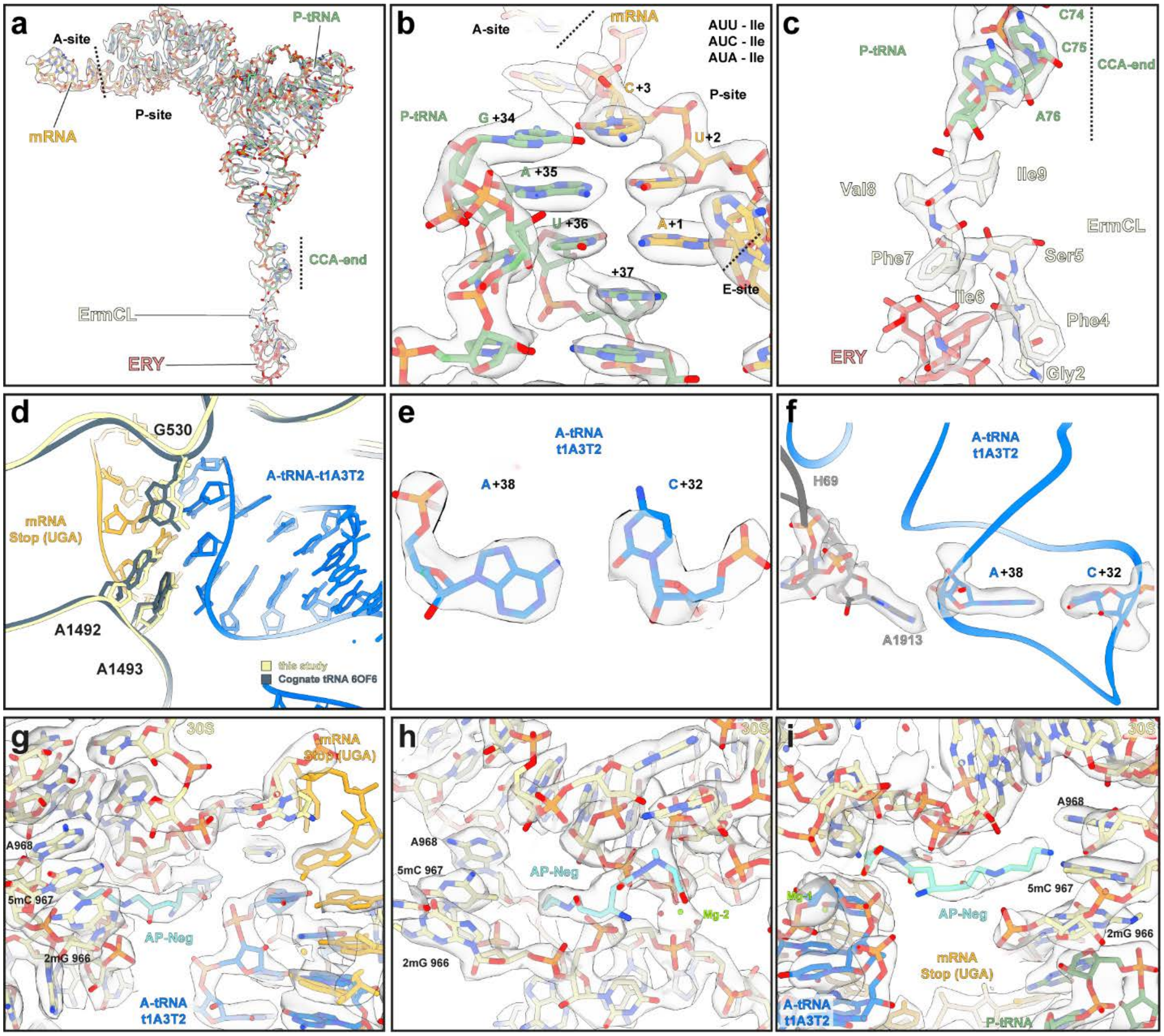

Supplementary Fig. 10. Density images for the 70S-UGA complex. (**a**) Isolated density (transparent grey) and model (green) for the ErmCL-peptidyl-tRNA in the P-site of the 70S-UGA complex. (**b**) Zoom showing the density (transparent grey) and model for the interaction between the Ile codon of the mRNA (orange) and the anticodon of the tRNA (green) in the P-site. (**c**) Zoom showing the density (transparent grey) and model for the CCA-end of the P-tRNA (green), ErmCL nascent chain (beige) and ERY (pink). (**d**) Conformation of G530, A1492 and A1493 (yellow) when t1A3T2 (blue) decodes UGA codon on mRNA (orange) on the ribosome compared with conformation of G530, A1492 and A1493 (dark green) when native tRNA^{Ala}GGC decodes a cognate GCC codon. (**e**) Density (transparent grey) and models (blue) for the C32-A38 base pair within the anticodon stem of the t1A3T2 tRNA. (**f**) Engaged conformation of A1913 (grey with transparent density) from H69 of the 23S rRNA with C32-A38 base pair of t1A3T2 tRNA (blue with transparent density). (**g-i**) Close-up views with density (transparent grey) of AP-Neg (cyan)

bound to helices h31 and h34 of the 30S subunit (yellow) in close proximity to the t1A3T2 tRNA (blue) and UGA stop codon of the mRNA (orange) in the A-site.

Supplementary Table 1. Sequences of the tRNA variants used in this study.

Predicted ΔΔG° values for substituting the TΨC-stem base pairs 49-65, 50-64 and 51-63 of *E. coli* $tRNA^{Phe}$ for other base pairs to estimate binding affinities to elongation factor EF-Tu[18]. ACC, acceptor; AC, anticodon; D and T, D- and TΨC-, respectively; s stem, l, loop. *P(S)* refers to the probability of each sequence to form a cloverleaf secondary structure assessed using the full equilibrium partition function (formula (16), ref.[59]). Nucleotide substitutions are highlighted: ACC (orange), D-s and D-l (light green), AC-s and AC-l (red), V-r (blue) and T-s and T-l (dark green).

Name	ACC-s		D-s	D-l	D-s		AC-s	AC-l	AC-s	V-r	T-s	T-l	T-s	ACC-s		CCA	ΔΔG° [kcal/mol]	*P(S)*
Position	1	8	10	14	22	26	27	32	39	44	49	54	61	66	73	74		
t1	GGGGCGG	UA	GCUC	AGAAGGGA	GAGC	A	GCGGA	GACUAAA	UCCGC	GAGAC	GGUCC	UUCGAUU	GGACC	CCGCUCC	A	CCA	-0.4	0.93
t1	GGGGCGG	UA	GCUC	AGAAGGGA	GAGC	A	GCGGA	GAUCAAA	UCCGC	GAGAC	GGUCC	UUCGAUU	GGACC	CCGCUCC	A	CCA	-0.4	0.93
t1A1	GGGGCGG	UA	GCUC	AGAAGGGA	GAGC	A	GCGGA	GUCUAAA	UCCGC	GAGAC	GGUCC	UUCGAUU	GGACC	CCGCUCC	A	CCA	-0.4	0.92
t1A1	GGGGCGG	UA	GCUC	AGAAGGGA	GAGC	A	GCGGA	GUUCAAA	UCCGC	GAGAC	GGUCC	UUCGAUU	GGACC	CCGCUCC	A	CCA	-0.4	0.92
t1A2	GGGGCGG	UA	GCUC	AGAAGGGA	GAGC	A	GCGGA	CUCUAGA	UCCGC	GAGAC	GGUCC	UUCGAUU	GGACC	CCGCUCC	A	CCA	-0.4	0.53
t1A2	GGGGCGG	UA	GCUC	AGAAGGGA	GAGC	A	GCGGA	CUUCAGA	UCCGC	GAGAC	GGUCC	UUCGAUU	GGACC	CCGCUCC	A	CCA	-0.4	
t1A3	GGGGCGG	UA	GCUC	AGAAGGGA	GAGC	A	GCGGC	CUCUAGA	GCCGC	GAGAC	GGUCC	UUCGAUU	GGACC	CCGCUCC	A	CCA	-0.4	0.48
t1A3	GGGGCGG	UA	GCUC	AGAAGGGA	GAGC	A	GCGGC	CUUCAGA	GCCGC	GAGAC	GGUCC	UUCGAUU	GGACC	CCGCUCC	A	CCA	-0.4	0.48
t1A4	GGGGCGG	UA	GCUC	AGAAGGGA	GAGC	A	GCGGC	CUUCAAA	GCCGC	GAGAC	GGUCC	UUCGAUU	GGACC	CCGCUCC	A	CCA	-0.4	
t1A5	GGGGCGG	UA	GCUC	AGAAGGGA	GAGC	A	GCGGC	UUUCAAA	GCCGC	GAGAC	GGUCC	UUCGAUU	GGACC	CCGCUCC	A	CCA	-0.4	
t1A6	GGGGCGG	UA	GCUC	AGAAGGGA	GAGC	A	GCGGC	UUUCAAC	GCCGC	GAGAC	GGUCC	UUCGAUU	GGACC	CCGCUCC	A	CCA	-0.4	
t1A3T1	GGGGCGG	UA	GCUC	AGAAGGGA	GAGC	A	GCGGC	CUUCAGA	GCCGC	GAGAC	UGCCC	UUCGAUU	GGGCA	CCGCUCC	A	CCA	-0.7	
t1A3T2	GGGGCGG	UA	GCUC	AGAA GGGA	GAGC	A	GCGGC	CUUCAGA	GCCGC	GAGAC	AGGGG	UUCGAUU	CCCCU	CCGCUCC	A	CCA	-1.5	
t1A3D	GGGGCGG	UA	GCGC	AGCCUGGUA	GCGC	A	GCGGC	CUUCAGA	GCCGC	GAGAC	GGUCC	UUCGAUU	GGACC	CCGCUCC	A	CCA	-0.4	
t1A3DT1	GGGGCGG	UA	GCGC	AGCCUGGUA	GCGC	A	GCGGC	CUUCAGA	GCCGC	GAGAC	UGCCC	UUCGAUU	GGGCA	CCGCUCC	A	CCA	-0.7	
t1A3DT2	GGGGCGG	UA	GCGC	AGCCUGGUA	GCGC	A	GCGGC	CUUCAGA	GCCGC	GAGAC	AGGGG	UUCGAUU	CCCCU	CCGCUCC	A	CCA	-1.5	

t1A3DT2 V1	GGGGCGG	UA	GCGC	AGCCUGGUA	GCGC	A	GCGGC	CUUCAGA	GCCGC	UGGGGUCACUCCCCG	AGGGG	UUCGAUU	CCCCU	CCGCUCC	A	CCA	-1.5	
t1A3DT2 V2	GGGGCGG	UA	GCGC	AGCCUGGUA	GCGC	A	GCGGC	CUUCAGA	GCCGC	UGGGGUCCACUCCCCG	AGGGG	UUCGAUU	CCCCU	CCGCUCC	A	CCA	-1.5	
t1A3T2 V1	GGGGCGG	UA	GCUC	AGAAGGGA	GAGC	A	GCGGC	CUUCAGA	GCCGC	UGGGGUCACUCCCCG	AGGGG	UUCGAUU	CCCCU	CCGCUCC	A	CCA	-1.5	
t1A3T2 V2	GGGGCGG	UA	GCUC	AGAAGGGA	GAGC	A	GCGGC	CUUCAGA	GCCGC	UGGGGUCCACUCCCCG	AGGGG	UUCGAUU	CCCCU	CCGCUCC	A	CCA	-1.5	
t2	GGGGCUC	UA	GCUC	AGAAGGGA	GAGC	A	GGGAC	GACUAAA	GUCCC	GAGAC	GGCGC	UUCGAUU	GCGCC	GAGCUCC	A	CCA	-0.9	0.88
t2AS1	GGGGCGC	UA	GCUC	AGAAGGGA	GAGC	A	GGGAC	GACUAAA	GUCCC	GAGAC	GGCGC	UUCGAUU	GCGCC	GCGCUCC	A	CCA	-0.9	
t2AS2	GGGGCUG	UA	GCUC	AGAAGGGA	GAGC	A	GGGAC	GACUAAA	GUCCC	GAGAC	GGCGC	UUCGAUU	GCGCC	CAGCUCC	A	CCA	-0.9	
t2AS2	GGGGCUG	UA	GCUC	AGAAGGGA	GAGC	A	GGGAC	GAUCAAA	GUCCC	GAGAC	GGCGC	UUCGAUU	GCGCC	CAGCUCC	A	CCA	-0.9	
t2AS3	GGGGCGG	UA	GCUC	AGAAGGGA	GAGC	A	GGGAC	GACUAAA	GUCCC	GAGAC	GGCGC	UUCGAUU	GCGCC	CCGCUCC	A	CCA	-0.9	
t2AS3	GGGGCGG	UA	GCUC	AGAAGGGA	GAGC	A	GGGAC	GAUCAAA	GUCCC	GAGAC	GGCGC	UUCGAUU	GCGCC	CCGCUCC	A	CCA	-0.9	
t2AS2 A3	GGGGCUG	UA	GCUC	AGAAGGGA	GAGC	A	GGGAC	CUUCAGA	GUCCC	GAGAC	GGCGC	UUCGAUU	GCGCC	CAGCUCC	A	CCA	-0.9	
t2AS2 T2	GGGGCUG	UA	GCUC	AGAAGGGA	GAGC	A	GGGAC	GAUCAAA	GUCCC	GAGAC	AGGGG	UUCGAUU	CCCCU	CAGCUCC	A	CCA	-1.5	
t2AS2 D	GGGGCUG	UA	GCGC	AGCCUGGUA	GCGC	A	GGGAC	GAUCAAA	GUCCC	GAGAC	GGCGC	UUCGAUU	GCGCC	CAGCUCC	A	CCA	-0.9	
t2AS2 A3T2	GGGGCUG	UA	GCUC	AGAAGGGA	GAGC	A	GGGAC	CUUCAGA	GUCCC	GAGAC	AGGGG	UUCGAUU	CCCCU	CAGCUCC	A	CCA	-1.5	
t2AS2 A3D	GGGGCUG	UA	GCGC	AGCCUGGUA	GCGC	A	GGGAC	CUUCACU	GUCCC	GAGAC	GGCGC	UUCGAUU	GCGCC	CAGCUCC	A	CCA	-0.9	
t2AS2 A3DT2	GGGGCUG	UA	GCGC	AGCCUGGUA	GCGC	A	GGGAC	CUUCACU	GUCCC	GAGAC	AGGGG	UUCGAUU	CCCCU	CAGCUCC	A	CCA	-1.5	
t3	GGGGCCC	UA	GCUC	AGAAAGGA	GAGC	A	GGCAG	GACUAAA	CUGCC	GAGAA	GCAGC	GACAUAA	GCUGC	GGGCUCC	A	CCA	-0.5	0.97
t4	GGGGCCC	UA	GCUC	AGAAAGGA	GAGC	A	GGCAG	GACUAAA	CUGCC	GAGAA	GCCGG	GACUAAA	CCGGC	GGGCUCC	A	CCA	-1.1	0.94
t5	GGGGCGC	UA	GCUC	AAUAAGGA	GAGC	A	GGAGC	GACUAAA	GCUCC	GAGAA	GUCGC	GACAUAA	GCGAC	GCGCUCC	A	CCA	-0.9	0.94
Ala(GGC)	GGGGCUA	UA	GCUC	AGCUGGGA	GAGC	G	CUUGC	AUGGCAU	GCAAG	AGGUC	AGCGG	UUCGAUC	CCGCU	UAGCUCC	A	CCA	-1.0	0.09
Ala(CUA)	GGGGCUA	UA	GCUC	AGCUGGGA	GAGC	G	CUUGC	AUCUAAU	GCAAG	AGGUC	AGCGG	UUCGAUC	CCGCU	UAGCUCC	A	CCA	-1.0	
Ala(UGC)	GGGGCUA	UA	GCUC	AGCUGGGA	GAGC	G	CCUGC	UUUGCAC	GCAGG	AGGUC	UGCGG	UUCGAUC	CCGCA	UAGCUCC	A	CCA	-0.7	0.005

Supplementary Table 2. Cryo-EM data collection, refinement and validation statistics

	t1A3T2 70S<br>EMDB-12035)<br>(PDB 7B5K)
**Data collection and processing**	
Microscope	Talos Arctica
Magnification	150,000
Voltage (kV)	200
Electron exposure (e^{-}/Å^2)	30
Defocus range (μm)	-0.8 to -3
Pixel size (Å)	0.96
Symmetry imposed	C1
Initial particle images (no.)	79,477
Final particle images (no.)	53,115
Map resolution (Å)	2.9
FSC threshold	0.143
Map resolution range (Å)	2.5-7.5
Detector	Falcon 3
**Refinement**	
Initial model used (PDB code)	6TBV
Map sharpening *B* factor (Å^2)	-46.13
Model composition	
Non-hydrogen atoms	148,612
Protein residues	5,684
RNA bases	4,790
Ligands	Ery, 2x AP-NEG
R.m.s. deviations	
Bond lengths (Å)	0.007
Bond angles (°)	1.037
Validation	
MolProbity score	1.84
Clashscore	4
Poor rotamers (%)	0.45
Ramachandran plot	
Favored (%)	95.95
Allowed (%)	3.95
Outliers (%)	0.11

Supplementary Table 3: Cloning primers, primers used for in vitro T7-based transcription of tRNA variants and oligonucleotides for probing. fw, forward primer, rev, reverse primer.

Name	Sequence (5′-3′)
**Primers for T7 transcription of the tRNA variants[a]**	
t1_fw	TAATACGACTCACTATAGGGGCGGTAGCTCAGAAGGGAGAGCAGCGGAGACTAAATCC
t1_rev	TGGTGGAGCGGGGTCCAATCGAAGGACCGTCTCGCGGATTTAGTCTCCGCTGCTCTCC
t1A1_fw	TAATACGACTCACTATAGGGGCGGTAGCTCAGAAGGGAGAGCAGCGG
t1A1_rev	TGGTGGAGCGGGGTCCAATCGAAGGACCGTCTCGCGGATTTAGACTCCGCTGCTCTCCCTTCTG
t1A2_fw	TAATACGACTCACTATAGGGGCGGTAGCTCAGAAGGGAGAGCAGCGG
t1A2_rev	TGGTGGAGCGGGGTCCAATCGAAGGACCGTCTCGCGGATCTAGAGTCCGCTGCTCTCCCTTCTG
t1A3_fw	TAATACGACTCACTATAGGGGCGGTAGCTCAGAAGGGAGAGCAGCGGCCTCTAGAGCC
t1A3_rev	TGGTGGAGCGGGGTCCAATCGAAGGACCGTCTCGCGGCTCTAGAGGCCGCTGCTC
t2_fw	TAATACGACTCACTATAGGGGCTCTAGCTCAGAAGGGAGAGCAGGGACGACTAAAGTCC
t2_rev	TGGTGGAGCTCGGCGCAATCGAAGCGCCGTCTCGGGACTTTAGTCGTCCCTGCTCTCC
t2AS1_fw	TAATACGACTCACTATAGGGGCGCTAGCTCAGAAGGGAGAGCAGGGACGACTAAAGTCC
t2AS1_rev	TGGTGGAGCGCGGCGCAATCGAAGCGCCGTCTCGGGACTTTAGTCGTCCCTGCTCTCC
t2AS2_fw	TAATACGACTCACTATAGGGGCTGTAGCTCAGAAGGGAGAGCAGGGACGACTAAAGTCC
t2AS2_rev	TGGTGGAGCTGGGCGCAATCGAAGCGCCGTCTCGGGACTTTAGTCGTCCCTGCTCTCC
t2AS3_fw	TAATACGACTCACTATAGGGGCGGTAGCTCAGAAGGGAGAGCAGGGACGACTAAAGTCC
t2AS3_rev	TGGTGGAGCGGGGCGCAATCGAAGCGCCGTCTCGGGACTTTAGTCGTCCCTGCTCTCC
t3_fw	TAATACGACTCACTATAGGGGCCCTAGCTCAGAAAGGAGAGCAGGCAGGACTAAAC
t3_rev	TGGTGGAGCCCGCAGCTTATGTCGCTGCTTCTCGGCAGTTTAGTCCTGCCTGCTCTC
t4_fw	TAATACGACTCACTATAGGGGCCCTAGCTCAGAAAGGAGAGCAGGCAGGACTAAAC
t4_rev	TGGTGGAGCCCGCCGGTTTAGTCCCGGCTTCTCGGCAGTTTAGTCCTGCCTGCTCTC
t5_fw	TAATACGACTCACTATAGGGGCGCTAGCTCAATAAGGAGAGCAGGAGCGACTAAAG
t5_rev	TGGTGGAGCGCGTCGCTTATGTCGCGACTTCTCGGAGCTTTAGTCGCTCCTGCTCTC
Ala(GGC)_fw	TAATACGACTCACTATAGGGGCTATAGCTCAGCTGGGAGAGCGCTTGCATGGCATGCAAG
Ala(GGC)_rev	TGGTGGAGCTAAGCGGGATCGAACCGCTGACCTCTTGCATGCCATGCAAGCGCTC
Ala(CUA)_fw	TAATACGACTCACTATAGGGGCTATAGCTCAGCTGGGAGAGCGCTTGCATCTAATGCAAG
Ala(CUA)_rev	TGGTGGAGCTAAGCGGGATCGAACCGCTGACCTCTTGCATTAGATGCAAGCGCTC
Ala(UGC)_fw	TAATACGACTCACTATAGGGGCTATAGCTCAGCTGGGAGAGCGCCTGCTTTGCACGCAG
Ala(UGC)_rev	TGGTGGAGCTATGCGGGATCGAACCGCAGACCTCCTGCGTGCAAAGCAGGCGCTCTC
**Oligonucleotide for CCA-end probing**	
CCA_probe	pCGCACUGCdTdTXdTdTdGdCdAdGdTdGdCdGdTdGdGdN, X= Cy3-dT
**Primers for tRNA variants cloning**	
t1_fw[b]	CGAGACGGTCCTTCGATTGGACCCCGCTCCACCAATCCTTAGCGAAAGCTAAGGATTTTTTTTAC
t1_rev[b]	CGGATTTAGTCTCCGCTGCTCTCCCTTCTGAGCTACCGCCCCAGCGTTACAAGTATTACACAAAGTTTTTATG
t1_fw[c]	TAACCGGAATTCGGGGCGGTAGCTCAGAAG
t1_rev[c]	CAATGCATTGGTTCTGCAGTGGTGGAGCGGGGTC
t1_fw[d]	CAGAAGGGAGAGCAGCGGAGATCAAATCCGCGAGACGGTCCTTCG
t1_rev[d]	CGAAGGACCGTCTCGCGGATTTGATCTCCGCTGCTCTCCCTTCTG
t1A1_fw	CAGAAGGGAGAGCAGCGGAGTTCAAATCCGCGAGACGGTCCTTCG
t1A1_rev	CGAAGGACCGTCTCGCGGATTTGAACTCCGCTGCTCTCCCTTCTG
t1A2_fw	CAGAAGGGAGAGCAGCGGACTTCAGATCCGCGAGACGGTCCTTCG
t1A2_rev	CGAAGGACCGTCTCGCGGATCTGAAGTCCGCTGCTCTCCCTTCTG
t1A3_fw	CAGAAGGGAGAGCAGCGGCCTTCAGAGCCGCGAGACGGTCC
t1A3_rev	GGACCGTCTCGCGGCTCTGAAGGCCGCTGCTCTCCCTTCTG
t1A4_fw	GCGGCCTTCAAAGCCGCGAG
t1A4_rev	CTCGCGGCTTTGAAGGCCGC
t1A5_fw	GAGCAGCGGCTTTCAAAGCCGC
t1A5_rev	GCGGCTTTGAAAGCCGCTGCTC
t1A6_fw	GCGGCTTTCAACGCCGCGAGAC
t1A6_rev	GTCTCGCGGCGTTGAAAGCCGC
t1A3T1_fw	GCCGCGAGACTGCCCTTCGATTGGGCACCGCTCCACCACTGCAGATCCTTAGCGAAAGCTAAG
t1A3T1_rev	TCTGAAGGCCGCTGCTCTCCCTTCTGAGCTACCGCCCCGAATTCAGCGTTACAAGTATTACACAAAGTTTTTATG
t1A3T2_fw	GCCGCGAGACAGGGGTTCGATTCCCCTCCGCTCCACCACTGCAGATCCTTAGCGAAAGCTAAG
t1A3T2_rev	TCTGAAGGCCGCTGCTCTCCCTTCTGAGCTACCGCCCCGAATTCAGCGTTACAAGTATTACACAAAGTTTTTATG
t1A3D_fw	GCCGCGAGACGGTCCTTCGATTGGACCCCGCTCCACCACTGCAGATCCTTAGCGAAAGCTAAG
t1A3D_rev	TCTGAAGGCCGCTGCGCTACCAGGCTGCGCTACCGCCCCGAATTCAGCGTTACAAGTATTACACAAAGTTTTTATG
t1A3DT1_fw	GCCGCGAGACTGCCCTTCGATTGGGCACCGCTCCACCACTGCAGATCCTTAGCGAAAGCTAAG
t1A3DT1_rev	TCTGAAGGCCGCTGCGCTACCAGGCTGCGCTACCGCCCCGAATTCAGCGTTACAAGTATTACACAAAGTTTTTATG
t1A3DT2_fw	GCCGCGAGACAGGGGTTCGATTCCCCTCCGCTCCACCACTGCAGATCCTTAGCGAAAGCTAAG
t1A3DT2_rev	TCTGAAGGCCGCTGCGCTACCAGGCTGCGCTACCGCCCCGAATTCAGCGTTACAAGTATTACACAAAGTTTTTATG
t1A3DT2 V1_fw	ACTCCCCGAGGGGTTCGATTCCCCTCC
t1A3DT2 V1_rev	GACCCCAGCGGCTCTGAAGGCCG
t1A3DT2 V2_fw	ACTCCCCGAGGGGTTCGATTCCCCTCC
t1A3DT2 V2_rev	GGACCCCAGCGGCTCTGAAGGCCG
t1A3T2 V1_fw	ACTCCCCGAGGGGTTCGATTCCCCTCC
t1A3T2 V1_rev	GACCCCAGCGGCTCTGAAGGCCG
t1A3T2 V2_fw	ACTCCCCGAGGGGTTCGATTCCCCTCC

t1A3T2 V2_rev	GGACCCCAGCGGCTCTGAAGGCCG
t2AS2_fw	CGATCAAAGTCCCGAGACGGCGCTTCGATTGCGCCCAGCTCCACCACTGCAGATCCTTAGCGAAAGCTAAG
t2AS2_rev	TCCCTGCTCTCCCTTCTGAGCTACAGCCCCGAATTCAGCGTTACAAGTATTACACAAAG
t2AS2 A3_fw	CCTTCAGAGTCCCGAGACGGCGCTTCGATTGCGCCCAGCTCCACCACTGCAGATCCTTAGCGAAAGCTAAG
t2AS2 A3_rev	TCCCTGCTCTCCCTTCTGAGCTACAGCCCCGAATTCAGCGTTACAAGTATTACACAAAG
t2AS2 T2_fw	CGATCAAAGTCCCGAGACAGGGGTTCGATTCCCCTCAGCTCCACCACTGCAGATCCTTAGCGAAAGCTAAG
t2AS2 T2_rev	TCCCTGCTCTCCCTTCTGAGCTACAGCCCCGAATTCAGCGTTACAAGTATTACACAAAG
t2AS2 D_fw	CGATCAAAGTCCCGAGACGGCGCTTCGATTGCGCCCAGCTCCACCACTGCAGATCCTTAGCGAAAGCTAAG
t2AS2 D_rev	TCCCTGCGCTACCAGGCTGCGCTACAGCCCCGAATTCAGCGTTACAAGTATTACACAAAG
t2AS2 A3T2_fw	CCTTCAGAGTCCCGAGACAGGGGTTCGATTCCCCTCAGCTCCACCACTGCAGATCCTTAGCGAAAGCTAAG
t2AS2 A3T2_rev	TCCCTGCTCTCCCTTCTGAGCTACAGCCCCGAATTCAGCGTTACAAGTATTACACAAAG
t2AS2 A3D_fw	CCTTCAGAGTCCCGAGACGGCGCTTCGATTGCGCCCAGCTCCACCACTGCAGATCCTTAGCGAAAGCTAAG
t2AS2 A3D_rev	TCCCTGCGCTACCAGGCTGCGCTACAGCCCCGAATTCAGCGTTACAAGTATTACACAAAG
t2AS2 A3DT2_fw	CCTTCAGAGTCCCGAGACAGGGGTTCGATTCCCCTCAGCTCCACCACTGCAGATCCTTAGCGAAAGCTAAG
t2AS2 A3DT2_rev	TCCCTGCGCTACCAGGCTGCGCTACAGCCCCGAATTCAGCGTTACAAGTATTACACAAAG
t2AS3_fw	CGATCAAAGTCCCGAGACGGCGCTTCGATTGCGCCCCGCTCCACCACTGCAGATCCTTAGCGAAAGCTAAG
t2AS3_rev	TCCCTGCTCTCCCTTCTGAGCTACCGCCCCGAATTCAGCGTTACAAGTATTACACAAAG
Ala(GGC)_fw	TTGCATGGCATGCAAGAGGTCAGCGGTTCGATCCCGCTTAGCTCCACCACTGCAGATCCTTAGCGAAAGCTAAG
Ala(GGC)_rev	GCGCTCTCCCAGCTGAGCTATAGCCCCGAATTCAGCGTTACAAGTATTACACAAAG
Ala(UGC)_fw	CTGCTTTGCACGCAGGAGGTCTGCGGTTCGATCCCGCATAGCTCCACCACTGCAGATCCTTAGCGAAAGCTAAG
Ala(UGC)_rev	GCGCTCTCCCAGCTGAGCTATAGCCCCGAATTCAGCGTTACAAGTATTACACAAAG
**Primers for GFP variants cloning**	
GFP_fw[e]	TAACGCTCTAGATTAACTTTAAGAAGGAGATATACCATG
GFP_rev[e]	TGCCCAAGCTTTTACTTGTACAGCTCGTCCAT
GFP(UAA)_fw	GCCACAAGTTCTAAGTGTCCG
GFP(UAA)_rev	CGGACACTTAGAACTTGTGGC
GFP(UAG)_fw	GCCACAAGTTCTAGGTGTCC
GFP(UAG)_rev	GGACACCTAGAACTTGTGGC
GFP(UGA)_fw	GCCACAAGTTCTGAGTGTCC
GFP(UGA)_rev	GGACACTCAGAACTTGTGGC
GFP_6xHis_fw	CACCATCACTAAAAGCTTGGCTGTTTTGGC
GFP_6xHis_rev	ATGGTGATGCTTGTACAGCTCGTCCATGC
**Oligonucleotide for affinity purification of t1A3T2**	
t1A3T2 probe	XTGGTGGAGCGGAGGGGAATCGAACCCCTGTCTCGCGGCTCTGAAG, X= Biotin

[a]T7 promoter sequence underlined

[b]t1(CUA) was cloned into pRIL vector, [c]subcloned into pBST NAV2, and [d]converted to t1(UCA)

[e]GFP was subcloned into pBAD33
