# Supplementary material for: Repurposing tRNAs for nonsense suppression
Source: Nat Commun. 2021 Jun 22;12:3850. doi: 10.1038/s41467-021-24076-x (PMC8219837; doi:10.1038/s41467-021-24076-x)
Supplement: Supplementary file 3 — Reporting Summary [file 41467_2021_24076_MOESM3_ESM.pdf]

nature research

Corresponding author(s): Zoya Ignatova

Last updated by author(s): May 24, 2021

Reporting Summary

Nature Research wishes to improve the reproducibility of the work that we publish. This form provides structure for consistency and transparency in reporting. For further information on Nature Research policies, see our Editorial Policies and the Editorial Policy Checklist.

Statistics

For all statistical analyses, confirm that the following items are present in the figure legend, table legend, main text, or Methods section.

n/a	Confirmed	
☐	☒	The exact sample size (n) for each experimental group/condition, given as a discrete number and unit of measurement
☐	☒	A statement on whether measurements were taken from distinct samples or whether the same sample was measured repeatedly
☐	☒	The statistical test(s) used AND whether they are one- or two-sided<br>*Only common tests should be described solely by name; describe more complex techniques in the Methods section.*
☒	☐	A description of all covariates tested
☒	☐	A description of any assumptions or corrections, such as tests of normality and adjustment for multiple comparisons
☐	☒	A full description of the statistical parameters including central tendency (e.g. means) or other basic estimates (e.g. regression coefficient) AND variation (e.g. standard deviation) or associated estimates of uncertainty (e.g. confidence intervals)
☐	☒	For null hypothesis testing, the test statistic (e.g. F, t, r) with confidence intervals, effect sizes, degrees of freedom and P value noted<br>*Give P values as exact values whenever suitable.*
☒	☐	For Bayesian analysis, information on the choice of priors and Markov chain Monte Carlo settings
☒	☐	For hierarchical and complex designs, identification of the appropriate level for tests and full reporting of outcomes
☒	☐	Estimates of effect sizes (e.g. Cohen's d, Pearson's r), indicating how they were calculated

Our web collection on statistics for biologists contains articles on many of the points above.

Software and code

Policy information about availability of computer code

Data collection	Flow cytometry: FACS Calibur (Becton Dickinson) with CellQuestTM Pro Software (v6.0; Becton Dickinson)<br>Mass spectrometry: ESI-Q-TOF maXis (Bruker Daltonik)<br>Radio flow detection: Beta-RAM 6 (Lab logic)<br>Bulk fluorescence measurements: Victor Nivo (PerkinElmer)<br>CryoEM data were collected using the EPU software v2.8 (FEI, Netherlands)
Data analysis	Flow cytometry data analysis: Flowing Draw v10.7.2.<br>Mass spectrometry data analysis: Data Analysis software Version 4.2 (Bruker Daltonik)<br>ViennaRNA package Version 2.3.4. for secondary structure analysis; Code for RNA sequence design is available at github (https://github.com/marcom/dss-opt).<br>Cryo-EM: RELION v3.0 and v3.1 with MotionCor2 v1.2.1, Gctf v1.06, and Gautomatch v0.56 for processing micrographs, picking particles, classification and refining cryo-EM maps. SPHIRE (SPARX v4.0) for filtering according to local resolution. Coot v0.8 and v0.92 and ISOLDE v1.1 for model building and Phenix (dev-2947-000) for model refinement and statistics. Figures were generated using Pymol v2.4, Chimera v1.14, and ChimeraX v1.0.

For manuscripts utilizing custom algorithms or software that are central to the research but not yet described in published literature, software must be made available to editors and reviewers. We strongly encourage code deposition in a community repository (e.g. GitHub). See the Nature Research guidelines for submitting code & software for further information.

Data

Policy information about availability of data

All manuscripts must include a data availability statement. This statement should provide the following information, where applicable:

- Accession codes, unique identifiers, or web links for publicly available datasets
- A list of figures that have associated raw data
- A description of any restrictions on data availability

The cryo-electron microscopy maps and the respective coordinates for electron-microscopy-based model have been deposited in the EMDataBank and Protein Data Bank with the accession codes EMD-12035 and PDB ID 7B5K, respectively. Code for RNA sequence design is available at github (https://github.com/marcom/dss-opt). All other data are available in the main text or the supplementary materials.

Field-specific reporting

Please select the one below that is the best fit for your research. If you are not sure, read the appropriate sections before making your selection.

☒ Life sciences ☐ Behavioural & social sciences ☐ Ecological, evolutionary & environmental sciences

For a reference copy of the document with all sections, see nature.com/documents/nr-reporting-summary-flat.pdf

Life sciences study design

All studies must disclose on these points even when the disclosure is negative.

Sample size	Sample size as stated in figure legends, determined empirically and similar to the most existing studies in the same field. Nostatistical method was used to predetermine the sample size.
Data exclusions	Usually no data were excluded, except micrographs with low estimated resolution or poorly fitted CTFs were excluded from further processing, as were particles that clustered into poorly defined classes during 2D and 3D classification.
Replication	Experiments were reproduced in independent experiments and using independent experimental methods. In each figure legends the number of independent biological replicates is stated.
Randomization	3D refinement in RELION, particles are randomly placed in one of two subsets. These subsets are maintained for CTF refinement. Otherwise, no randomization was performed.
Blinding	No blinding was performed as blinding is not possible or not applicable for the experiments.

Reporting for specific materials, systems and methods

We require information from authors about some types of materials, experimental systems and methods used in many studies. Here, indicate whether each material, system or method listed is relevant to your study. If you are not sure if a list item applies to your research, read the appropriate section before selecting a response.

Materials & experimental systems

n/a	Involved in the study	
☐	☒	Antibodies
☒	☐	Eukaryotic cell lines
☒	☐	Palaeontology and archaeology
☒	☐	Animals and other organisms
☒	☐	Human research participants
☒	☐	Clinical data
☒	☐	Dual use research of concern

Methods

n/a	Involved in the study	
☒	☐	ChIP-seq
☐	☒	Flow cytometry
☒	☐	MRI-based neuroimaging

Antibodies

Antibodies used

- Anti-GFP from mouse IgG1κ
Roche
Catalog number: 11814460001
clones 7.1 and 13.1

- GAPDH Loading Control Monoclonal Antibody (GA1R), HRP
Thermo Fisher Scientific
Catalog number: MA5-15738-HRP

	Immunstar Goat anti-Mouse-HRP<br>Bio-Rad Laboratories<br>Catalog number: 170-5047
Validation	-Anti-GFP from mouse IgG1κ<br>Western Blot 1:1000<br>https://www.sigmaaldrich.com/catalog/product/roche/11814460001?lang=en&region=GB<br>See Fig. 3, Extended Data Fig. 2A, 3 and 5A<br><br>- GAPDH Loading Control Monoclonal Antibody (GA1R), HRP<br>Western Blot 1:500-1:2000<br>Species: Bacteria, Chicken, Hamster, Human, Insect, Mouse, Rabbit, Rat, Yeast<br>https://www.thermofisher.com/antibody/product/GAPDH-Loading-Control-Antibody-clone-GA1R-Monoclonal/MA5-15738-HRP#!#references-heading<br><br>-Immunstar Goat anti-Mouse-HRP<br>https://www.bio-rad.com/en-us/sku/1705047-immun-star-goat-anti-mouse-gam-hrp-conjugate?ID=1705047

Flow Cytometry

Plots

Confirm that:

☒ The axis labels state the marker and fluorochrome used (e.g. CD4-FITC).

☒ The axis scales are clearly visible. Include numbers along axes only for bottom left plot of group (a 'group' is an analysis of identical markers).

☒ All plots are contour plots with outliers or pseudocolor plots.

☒ A numerical value for number of cells or percentage (with statistics) is provided.

Methodology

Sample preparation	The pBST NAV2 bearing different tRNAs and pBAD33 encoding GFP UGA were cotransformed in E. coli XL1-blue cells and grown in LB-medium containing ampicillin (100 µg/mL) and chloramphenicol (34 µg/mL). At OD600nm of 0.4, GFP expression was induced with 0.05% or 0.25% L-arabinose and cells were further cultivated till OD600nm 1.0. Cells were pelleted by centrifugation, washed with phosphate buffered saline (PBS) and resuspended in PBS.<br>For natural readthough (supplemenatry fig. 2), the GFP stop-codon variants (UAA, UAG, UGA) were treated the same except induction for 2h.
Instrument	FACS Calibur (Becton Dickinson)
Software	CellQuestTM Pro Software (v6.0; Becton Dickinson) was used to collect the data.<br>Flowdraw v10.7.2 was used to analyze the data.
Cell population abundance	Appr. 100,000 events after gating were acquired. No post-sorting measurements were perfomed.
Gating strategy	Intact cells were gated using the log plot of SSC-H (y-Axis) against FSC-H (x-Axis). In mock-transformed sample (suppl. fig. 2c and 5) the same gate was applied to all samples within one biological replicate

☒ Tick this box to confirm that a figure exemplifying the gating strategy is provided in the Supplementary Information.
